# The development and role of microbial-host interactions in gut mucosal immune development

**DOI:** 10.1186/s40104-016-0138-0

**Published:** 2017-01-26

**Authors:** C. R. Stokes

**Affiliations:** School of Clinical Veterinary Science, University of Bristol, Langford House, Langford, Nr Bristol, BS40 5DU UK

**Keywords:** Gut microbiota, Intestine, Mucosal immune development, Pig, Rearing environment

## Abstract

At birth the piglet’s immune system is immature and it is dependent upon passive maternal protection until weaning. The piglet’s mucosal immune system develops over the first few weeks but has not reached maturity at weaning ages which are common on commercial farms. At weaning piglets are presented with a vast and diverse range of microbial and dietary/environmental antigens. Their ability to distinguish between antigens and mount a protective response to potential pathogens and to develop tolerance to dietary antigens is critical to their survival and failure to do so is reflected in the high incidence of morbidity and mortality in the post-weaning period. A growing recognition that the widespread use of antibiotics to control infection during this critical period should be controlled has led to detailed studies of those factors which drive the development of the mucosal immune system, the role of gut microbiota in driving this process, the origin of the bacteria that colonise the young piglet’s intestine and the impact of rearing environment. This review briefly describes how the mucosal immune system is equipped to respond “appropriately” to antigenic challenge and the programmed sequence by which it develops. The results of studies on the critical interplay between the host immune system and gut microbiota are discussed along with the effects of rearing environment. By comparing these with results from human studies on the development of allergies in children, an approach to promote an earlier maturation of the piglet immune system to resist the challenges of weaning are outlined.

## Background

The mucosal immune system that is associated with the gastrointestinal tract is essential both for protection from enteric infection and for many of the other physiological roles required of the gut for the maintenance of health and development. The gastrointestinal tract is a major interface between a host and it’s environment and whilst the epithelial layers of other interfaces, such as the skin, are well suited to prevent the absorption of harmful antigens, the gut is highly specialised for digestion and the absorption of nutrients. Although a recent study has calculated that the mean total mucosal surface of the digestive tract averages 32 m^2^ in man [1], approximately ten fold less than earlier estimates, it provides an interface that is ideal for a nutritional role but less so for preventing the entry of potential pathogens or their products. The gut mucosal environment is complicated by both the magnitude of challenge and the complex array of antigens that are presented and the immune system that is associated with the gastrointestinal tract is required to recognise these different groups of antigens and respond “appropriately”. For example in the human intestine the microbial component including luminal or mucosal-associated bacteria is composed of 6–10 *phyla* and approximately 5,000 different species [2]. The density of luminal bacteria increases along the gastrointestinal tract, reaching up to 10^12^ per gram of mucus in the colon. Perhaps not surprisingly within this vast microbial population are both commensals (which play an important role in host defence and drive immune development) and potential pathogens. The gut mucosal immune system is therefore required not only to distinguish between microbial and dietary antigens, but also between commensal and potentially pathogenic organisms.

There is a considerable body of evidence that the immune system of neonates is functionally different from that of adults [3–6]. The young animal is then highly dependent upon maternal passively derived immunity for their survival through this vulnerable “learning” period [7, 8]. There are a number of factors that drive the development of the mucosal immune system including maternally-derived antigen and antibody [5], maternal environment, host genotype, diet and the gut microbiome [9]. This developmental process is of pivotal importance and it has been reported that stress associated with early weaning of piglets (16–18 d) leads to an impaired innate mucosal immune response and increased susceptibility to challenge with enterotoxigenic *E. coli* challenge compared with those weaned at 20 d [10]. Interestingly it has been shown in adult rodents that social stress can also alter the intestinal microbiota community structure [11]. In species of agricultural importance such as the pig, antimicrobials are widely used to compensate for the piglets immature immune system, in order to control enteric infections. The widespread use of antimicrobials is now a major concern both in terms of the rapid increase in the spread of resistance to many antibiotics [12] and also in pollution of the environment with heavy metals [13]. The aim of this review is then to briefly discuss the structure and function of the adult gut mucosal immune system, the process of development of mucosal immunity in the young animal, and, in an agricultural context, factors that may drive/speed up this development process. With apologies to those who study “other species” much data summarised in this article will be based upon studies carried out in the pig.

### Architecture of the gut mucosal immune system

The anatomy and environment within which the mucosal immune system operates forms an integral part of its activity [14]. The mucus layer, together with anti-microbial peptides such as α-defensins released by Paneth cells, collectively forms the glycocalyx which traps invading micro-organisms and enables their expulsion. This process is facilitated by peristaltic movement. Below the glycocalyx, is the intestinal epithelium which includes several cell types, the vast majority of which are absorptive enterocytes, but also includes goblet cells, neuroendocrine cells and Paneth cells [15]. Mucosal “barrier function” is central to mucosal defence and is made up of a number of elements. Small intestinal epithelial cells arise from progenitor stem cells located in the crypts. As they migrate up the crypt and then villus, these cells mature and differentiate, changing from immature secretory cells to mature absorptive cells. Cells reaching the tips of the villus are then shed into the gut lumen. Importantly this occurs before the epithelial cells become effete, so avoiding any compromise to barrier function. Continuity of the barrier between adjacent epithelial cells is maintained through a series of specialised interactions made up of “tight junctions”, adherens junctions and desmasomes [16].

The traditional description of mucosal lymphoid tissue distinguishes between organised and diffuse lymphoid tissues. The organised lymphoid tissues associated with the intestine include the Peyer’s patches and mesenteric lymph nodes. The diffuse epithelial and connective tissue of the gut contains large numbers of leucocytes and it has been estimated that as many as 7% of all leucocytes are found in this site. In mammals, three compartments can be identified within the diffuse immunological areas. These include the epithelial compartment and the lamina propria of both the villi and crypts [17].

The lamina propria is well supplied with leucocytes and in contrast to many other species the immunological organisation of the lamina propria in the pig intestine shows a high level of organisation. Within the villus lamina propria the tissue deep to the capillary plexus contains predominantly CD4^+^ T cells whilst CD8^+^ cells occur luminally and in the epithelium [18, 19]. Antigen-presenting cells expressing MHC II are present in large numbers in the lamina propria of many species and in adult pigs, they have been characterised as functional, immature, dendritic cells [20]. The lamina propria around the intestinal crypts contains cells staining for immunoglobulins (predominantly IgA, presumably plasma cells), small numbers of T-cells and dendritic cells, and myeloid cells with the characteristics of macrophages and granulocytes.

At birth only small numbers of leucocytes are found in the lamina propria and in conventional pigs it becomes populated according to a clearly staged time course [21–24]. Within the first week dendritic cells which are strongly MHC II+ and co-express CD45and CD16 along with other myeloid markers appear. Initially, a subset expresses CD14 but in older animals this is lost, suggesting that at least some of these dendritic cells may be derived from blood monocytes. In contrast, T-cells appear more slowly and undergo a phased pattern of appearance [21]. An unusual cell type, characterised by the expression of CD2 and CD3, but lacking CD4 and CD8 (CD4^−^CD8^−^ T-cells),together with a second T-cell population, characterised as CD2^+^CD3^+^CD4^−^CD8αα^+^, form the dominant population of T-cells migrating into the jejunal tissue during the first week to ten days, and, which can still be found in adult animals, albeit in reduced proportions. Interestingly, whilst conventional CD4^+^ and CD8αβ^+^ T-cells in the lamina propria of adult animals express low levels of CD45RC, consistent with advanced memory status, there are a significant proportion of “unusual” CD2^+^CD3^+^CD4^−^CD8αα^+^ T-cells which express moderate to high levels of CD45RC, suggesting that they may be less antigen-experienced. During the first week to 10 days of life the level of CD25 expression on lamina propria cells is high, further indicating that they arrive with, or acquire an activated status in the intestinal wall of very young animals [20, 25].

During the second and third week of life, increasing numbers of “recently activated” CD4^+^ T-cells can be found in the lamina propria pool of T cells. This contrasts with the cellular characteristics of CD4^+^ T-cells in older animals, which by phenotype are resting cells, but of advanced memory status and which respond to polyclonal activation by expression of IL-4 mRNA but not IL-2. Significant numbers of cytotoxic T-cells, characterised by high levels of CD8 are present from the third week of life, although, a small proportion of such cells can be found as early as the first week. Other late arrivals in the gut are IgA^+^ plasma cells, which have been reported to appear in significant numbers as late as 3–6 wks. The “final architecture” of the diffuse lymphoid tissue of the gut is not achieved until the pig is approximately 6 weeks old, and includes large numbers of dendritic cells and CD4^+^ T-cells of resting, advanced memory phenotype, that can transcribe IL-4 but are unable to secrete IL-2 and respond to further activation by apoptosis [26].

### Antigen uptake & induction of mucosal responses

The structure of the mucosal immune system has been extensively reviewed [14, 15] and much attention has been focussed upon the role of Peyers patches and the mesenteric lymph nodes in the sampling and recognition of luminally-presented antigens. Several pathways have been described [27], most notably antigen can be sampled and transported through the specialised M-cells in the follicle associated epithelium that overlie the dome region of the Peyers patches. Antigen taken up by M –cells or paracellularly is transferred to dendritic cells in the dome of the patch and migration of these cells to the T-cell zones results in T-cell activation, migration and induction of responses in the follicle. Primed T- and B-cells emigrate from the patches in efferent lymphatics [10]. Secondly, antigen may be taken up across the epithelium of the villi outside the Peyers patches. In many species, cells of the dendritic lineage lie immediately underneath the intestinal epithelium and may acquire and transport antigen from several sources. They may extend dendrites through the epithelium by manipulating tight-cell junctions, allowing direct antigen sampling and bacterial trapping [28, 29]; they may acquire antigens which have crossed the epithelium intact, either transcellularly or paracellularly [30, 31]; or they may phagocytose epithelial cells, together with any environmental antigens which they may have acquired [32]. Following antigen acquisition, mucosal dendritic cells migrate through afferent lymphatics to the mesenteric lymph nodes, where they can present antigen in T-cell areas [32]. The presence of this pathway has led to acceptance that the mesenteric lymph nodes are important sites for initiation or expansion of mucosal immune responses [33]. Thirdly, intact antigen absorbed across the mucosal epithelium (either the villi or the Peyers patches) may reach the lymphatics directly and be transported to the lymph nodes and ultimately into blood, where it can interact with components of the systemic immune system including the spleen and distant lymph nodes [34]. Finally, antigen may be released from enterocytes in the form of ‘exosomes’. These subcellular structures have been described in human and rodent and consist of membrane bound MHC class II apparently complexed with antigen [35, 36]. The role of such structures in the pig is unclear as unlike humans and rodents they appear not to express MHC class II on their gut enterocytes [19]. In further contrast the venous capillary epithelium in the intestinal lamina propria of the pig expresses high levels of MHC class II molecules, and it may be that these cells release exosomes directly into blood.

### Induction of responses and homeing

The two key reasons that underlie the need for a better understanding of mechanisms that operate at mucosal surfaces are, an ability to control infections through the development of mucosal vaccines and, the protection from allergic reactions to otherwise harmless antigens through the development of oral tolerance. There is a large body of data to show that immune responses which are protective at mucosal surfaces are most effectively stimulated by local application of antigen [37], however the expression of active immune responses against antigens presented to the mucosa is frequently disadvantageous. The induction of an immune response requires the mobilisation of energy and resources from other activities (eg nutrition & growth). For example it was estimated that the response to infection requires a 20–25% increase in protein and amino acid usage [38]. The “protective” effector mechanisms of immune responses frequently result in tissue damage that is independent of that generated by the pathogen. Presumably, the temporary disadvantage of expression of an immune responses outweighs the long-term disadvantage of having to live, or die, with the pathogen. Since the diversity of challenge posed by antigens presented to the gut immune system varies from severe (e.g. pathogenic micro-organism) to low or absent (true commensal flora, food), this requires an ability to modulate responses that reflects the perceived threat, rather than simply the antigenic load. That is, the magnitude and type of response should be dependent on the ‘quality’ of the antigen, not solely on the quantity. In the case of most food antigens in normal individuals, this would, ideally, involve complete absence of immune responses or ‘immunological tolerance’. Oral tolerance is a specific acquired mechanism whereby prior feeding reduces an individual’s ability to respond to subsequent presentation of that antigen. The induction of oral tolerance has been very extensively studied in rodents and a number of regulatory process characterised. Following feeding, small quantities of fed protein (<0.02%) are absorbed intact across the intestinal mucosa. Whilst such levels may not be nutritionally significant, immunologically they are highly important and capable of eliciting both humoral and cellular immune responses that are comparable to that induced by injection [6].

The absorption of intact proteins from the diet raises the potential of eliciting damaging allergic reactions and food allergy. In order to prevent tissue damaging allergic responses to harmless dietary components these responses must be controlled and two regulatory mechanisms have been identified. The first involves the local production and secretion of IgA antibody into the intestinal mucus layer, where it may reduce the subsequent absorption of that dietary protein. This process has been termed “immune exclusion” [39]. This process is rarely absolute [40] and systemic tolerance to fed proteins (“oral tolerance”) may develop. In contrast to the response to injected antigens, which prime for a secondary response of greater magnitude than the primary response, feeding, after a transient primary response, normally leads to the development of oral tolerance. The latter being defined as specific acquired mechanism whereby prior exposure reduces an individual’s ability to respond to subsequent presentation of that antigen. A number of different mechanisms have been implicated in oral tolerance including active regulation by Foxp3^+^ regulatory T cells (Tregs), clonal deletion and clonal anergy [27]. Mucosally induced tolerance provides protection from the damaging allergic responses responsible for eczema, asthma, hay fever and food allergy. Whilst eczema, asthma and hay fever are not considered a problem in pig production, several years ago we presented data that led to the hypothesis that a transient allergic immune response to dietary antigen (prior to the induction of tolerance) might predispose to post-weaning diarrhoea in piglets [41].

In order to mount an effective mucosal immune response, cells are required to traffic between inductive (Peyer’s patch) and effector sites (lamina propria and epithelium). Naïve T cells are primed in the Peyer’s patches and migrate out from the gut via the mesenteric lymph node and thoracic duct, before homing back to the intestinal lamina propria. Lymphoid effector cells re-enter the circulation and return to the lamina propria through altered integrin and chemokine receptor expression. The migratory pathway requires the interaction between the ligand α4β7 (expressed by “mucosal lymphocytes”) and the mucosal cell addressin molecule, MAdCAM-1, which is expressed on vascular endothelium in mucosal tissues. Whilst the expression α4β7 has been associated with the homing of cells to the lamina propria, another member of the β7 subfamily of integrins has been implicated in the localisation of IEL’s. In the small intestine, lamina propria T cells are distributed primarily in the upper villus with gradually decreasing numbers to the crypts. In contrast the majority of B cells and plasma cells are present within the crypts with many fewer cells within the villus. Within the crypts in the small intestinal lamina propria the number of IgA producing plasma cells greatly exceeds those expressing IgG and IgM. The polymeric immunogloblin receptor (pIgR), which is required for the selective transport of locally synthesised IgA across epithelial cells into the gut lumen, is also largely restricted to the crypt region.

### Host – microbiota cross-talk

Over several years there has been a growing realisation of the importance of a cross-talk between the host immune system and the microbiota that inhabits the intestinal tract. It is well recognised that whilst the host immune system can regulate the interactions between the host and the gut microbiome [42] there is now a large body of evidence obtained from several species to show that gut microbiota drives the development and function of the mucosal immune system [43–45]. As described above the intestinal immune system can be divided into inductive (Peyer’s patches, isolated lymphoid follicles and mesenteric lymph nodes) and effector (lamina propria and epithelium) sites. The epithelium has the important immunologic function of transporting immunoglobulin (Ig) A into the lumen using the polymeric Ig receptor, and can also produce anti-microbial peptides, cytokines and chemokines in response to bacterial and viral invasion. Epithelial cells express pattern recognition receptors (PRRs), which are specialized in the interaction with conserved microbial products structures commonly referred to as pathogen-associated molecular patterns (PAMPs) [46]. PRRs comprise a group of transmembrane proteins, the toll-like receptors (TLRs), and a class of intracellular proteins, the nucleotide-binding oligomerisation domain (NOD)-like receptors (NLRs), which play a key role in microbial recognition [47, 48] and in the control of adaptive immune responses towards commensal and pathogenic bacteria.

In mammals, the TLRs comprise a family of 11 individual type I transmembrane receptors which are characterised by three common structural features: a divergent ligand-binding extracellular domain with leucine-rich repeats (LRRs), a short transmembrane region, and a highly homologous cytoplasmic Toll/interleukin (IL)-1 receptor (TIR) domain. TLRs are differentially (inducibly or constitutively) expressed by many distinct cell types throughout the whole GI tract, including gut epithelial cells, dendritic cells, macrophages, B cells, and T regulatory (Treg) cells [49]. Several PAMPs selectively activate specific PRRs. For example TLR4 recognizes bacterial lipopolisaccharide (LPS), TLR2 in combination with TLR1 or TLR6 recognize diacetylated or triacetylated bacterial lipopeptides respectively, TLR5 recognize flagellin and within endosomal vesicles TLR9 recognizes microbial DNA sequences that are rich in CpG motifs. The engagement of a TLR with its microbial ligand activates several signalling pathways, such as the NF-kB and the mitogen-activated protein kinase (MAPK) cascades. This results in the transcription of genes, necessary to mount a protective response against an invading microbial agent.

The NLR’s, which include two subfamilies called NODs and NALPs comprise of more than 20 cytoplasmic proteins that regulate inflammatory and apoptotic responses. They contain three distinct functional domains: a carboxy-terminal LRR domain which mediates ligand recognition, a centrally located nucleotide binding domain (NBD), and a structurally variable amino-terminal effector-binding domain which consists of protein-protein interaction domains, such as caspase recruitment domains (CARDs) or pyrin domains [50]. NOD1 recognizes a molecule called meso-DAP, which is a constituent of Gram negative bacteria. NOD2 proteins recognize intracellular MDP (muramyl dipeptide), which is a peptidoglycan constituent of both Gram positive and Gram negative bacteria.

### Factors that influence development

As described above the piglet is profoundly immunologically deficient at birth, being highly dependent upon maternally derived colostrum and milk for their early survival. The mucosal immune system develops in a programmed sequence but both phenotypically [24] and functionally [5, 6] there remain significant differences from that found in adults at standard commercial weaning ages. Whilst the sequence of development may be programmed there is a growing body of evidence to suggest that the rate of development may be determined by a range of host and environmental factors. The familial basis for human allergic diseases is well established [51]. We have shown using inbred strains of mice that there is gentic heterogenity in the development of tolerance to novel dietary antigens [52] and our preliminary studies in commercial lines of pigs that there are phenotypic differences in the development of the piglets mucosal immune system that might indicate that there are similar genetic differences. (data in preparation for publication).

#### Rearing environment: effect of high versus low hygiene conditions

There is a growing body of evidence to suggest that early rearing environment can profoundly affect an individual’s susceptibility to disease [53]. For example epidemiological studies have shown that children who grow up on traditional farms are protected from asthma, hay fever and allergic sensitization [54, 55]. Further studies have indicated that farm living leads to a modulation of innate and adaptive immune responses by intense microbial exposures delivered before or soon after birth [56]. Increasing evidence suggests that early life exposure to microbial flora drives expansion of the immune system [2], but that development of “specific arms” of the immune system may require colonisation with particular intestinal microbiota (for review see [43]). Given the evidence for the influence of early-life microbial colonisation on immunological development, we hypothesized that rearing piglets under “high or low hygiene conditions” would affect the functional development of mucosal immunity. Using this approach we have attempted to address the origin of the bacteria that colonise the young piglets, the critical period of exposure to bacteria, and the effect of magnitude and diversity of microbial challenge.

In the first series of experiments we investigated the effect of bacterial origin on long term carriage. Four different litters of conventionally reared inbred piglets born within 24 h of each other were kept with and allowed to suckle their own “mothers” for 28 d. Piglets were then weaned and “mixed” by allocating them into 5 different pens where then were housed for three more weeks. Piglets were then killed and gut microbiota analysed by DGGE, and the results analysed by non-metric, multidimensional scaling allocating individual piglets according to both litter and pen. The results show that whilst there is no evidence of clustering according to pen, there is clear clustering according to litter. This clearly shows that the microbiota acquired during the first 4 weeks of life profoundly influences the long term enteric carriage into the post-weaning period and later life. Whilst these results would not eliminate a contributing role of genotype since they were obtained in inbred Babrahams that share exactly the same genotype they highlight the importance of early life environment in determining the longer term carriage of enteric bacteria. Bacteria contributing to this micro-environment will be likely to have originated from the sow and her farrowing area [data in preparation for publication].

Under highly controlled conditions in which piglets were derived by caesarean section into fully germ-free bubbles, the effect colonisation with a defined, three-component microbiota was compared with littermates that remained “germ-free”. Colonisation resulted in expansion and development of the B-cell, T-cell and antigen-presenting-cell compartments of the mucosal immune system [26, 57],with differences in antigen-presenting cells apparent by 5 days old, whilst differences in T-cell compartments were not significant until 21 days old, suggesting either that effects on T-cells were mediated through initial effects on antigen-presenting cells, or that direct effects on CD4+ T-cells require more prolonged contact with microbiota.

Given the evidence for the influence of early-life microbial colonisation on immunological development, we hypothesized that rearing piglets under different conditions – either low hygiene (allowed to suckle from the sow) or high hygiene (formula fed) – would affect the functional development of mucosal immunity. Therefore, we examined the impact of alternative rearing conditions during the first month of life on intestinal microbiota, antigen presenting cell (APC) phenotype and T cell function in intestines from neonatal piglets reared under low and high hygiene conditions. We also investigated whether the farm of origin of the piglets (indoor-intensive versus outdoor-extensive) influenced the outcome of the development process and the important question as to how long the period of exposure to a particular environment was required in order to influence the outcome.

Over the past decade we have established an experimental model which allows us to compare piglets reared in high-containment, SPF isolators with their litter-mates reared on the sow under conventional husbandry conditions. To examine the effects of rearing environment, 12 piglets from six litters were matched into two equal groups 24 h after birth. One group (high hygiene) was removed to an SPF facility (positive-pressure, HEPA-filtered air), individually housed and automatically fed hourly with a commercial bovine milk formula. Litter matched siblings were left on the farm and were nursed by their mother (low-hygiene). The microbiota which initially establishes in isolator and sow-reared piglets is very similar but begins to diverge after 12 d [26]. By 28 and 56 days old, there are marked differences between isolator and farm-reared piglets both in their microbiota and in expression of a range of genes associated with innate immunity [58–60]. Dendritic cells accumulated in the intestinal mucosa in both groups, but more rapidly in isolator piglets. Importantly, outlier piglets whose microbiota changed early also accumulated dendritic cells earlier than the remainder of the group. Consistent with dendritic cell control of T-cell function, the effects on T-cells occurred at later time points, and mucosal T-cells from high-hygiene, isolator pigs made less IL-4 while systemic T-cells made more IL-2 [26]. We recognized that within our basic model a combination of factors, such genetics, diet, stress associated from maternal separation may also be influencing either singularly or in combination with gut microbiota the development of the gut mucosal immune system. We therefore used an increasingly reductionist approach to control for these variables. Whereas the initial studies directly compared isolator versus sow reared piglets in subsequent studies we compared isolator reared piglets, treated or not treated with antibiotics, born on either indoor or outdoor farms.

In the next set of experiments we studied the effect of birth environment (farm of origin: indoor versus outdoor), subsequent rearing environment and antibiotic treatment on the general CD4^+^ T cell population and on the CD4^+^CD25^+^Foxp3^+^ regulatory T cells. At 28 d after birth, piglets that were transferred to an isolator from the indoor farm had significantly fewer lamina propria CD4^+^CD25^+^Foxp3^+^ Tregs in comparison to their siblings that stayed with their mothers on the farm. Treatment with antibiotics did not reduce this number any further. In contrast there was no reduction in the number of Tregs in piglets transferred to the isolator from the outdoor farm, suggesting that 24 h on the outdoor farm was sufficient to maintain the Tregs cell population. Interestingly this “stimulatory effect” of 24 h on the outdoor farm was greatly reduced by treating the isolator piglets with antibiotics thus strongly implicating the gut microbiota in this protective role [61].

In order to test the hypothesised beneficial effects of Tregs, the response to a dietary antigen (introduced at weaning) was compared between farm reared piglets from an indoor unit (high levels of Tregs) with those reared in the isolator (low numbers of Tregs). The results showed that the increases in both serum IgG1 and IgG2 anti-soya antibody levels were significantly greater in the isolator reared pigs when compared with littermates that remained on the farm for the first 4 weeks of life strongly suggesting that early that the early rearing environment (and possibly the number of Tregs) significantly impacts upon the piglets’ ability to respond to antigens in the post-weaning diet ([61] and in preparation for publication). The newly weaned piglet is required to respond appropriately to a wide range of dietary and microbial antigens and we have postulated that failure to make such responses may predispose to postweaning diarrhoea.

The data on the effects of rearing environment on Tregs strongly indicates that microbial colonisation within the first 24 h of life to be of particular importance. It was important then to test if other lamina propria cell populations were similarly affected. Using quantitative fluorescence immunohistology we quantitated the expression of CD14, CD16, MHCII and MIL11 in the intestinal lamina propria. Data was subjected to Principal Component Analysis (PCA) with 16 combinations of proportional, cross-correlated areas of staining for the four markers. The PCA identified five orthogonal variables, explaining 84% of the variance. These represented: 1 = CD14 (LPS receptor), 2 = MIL11 + MHCII (endothelial cell presentation), 3 = CD16 + MIL11 + MHCII, 4 = MIL11 + CD16 (macrophage presentation), 5 = CD16 (low affinity Fc receptor). The analysis showed that the derived factors distinguish the effects of very early environment (i.e. born on an indoor or outdoor farm) factor 3 (CD16, MIL11, MHCII) and later rearing (kept on the sow, transferred to an SPF isolator of transferred to an SPF isolator and treated with antibiotics), factors 2 & 4. Together these results highlight the importance of the vascular endothelium as the primary target for the effects of early-life environment [62].

### What do studies in humans tell us?

It was reported many years ago that children born to parents with a uni-lateral (56%) or bi-lateral (72%) family history of allergic disease are more likely to go on and develop allergies (eczema, asthma or hay fever) than children born to non-allergic parents [51]. It was also shown that a transient deficiency in IgA at months of age may predict the onset of allergy during the first year of life, suggesting early life experience may have a critical effect in determining later disease onset [63]. Early studies also showed that allergen avoidance (including breast feeding) during the first few months of life could have a beneficial effect in reducing the number of children who became allergic. Although other studies have not always been able to replicate these clinical observations there is clear evidence to show that exclusively formula-fed infants were more often colonized with *E coli*, *C difficile*, *Bacteroides*, and lactobacilli, compared with breastfed infants [64]. The same authors showed that at 1 month of age infants born through cesarean section had lower numbers of bifidobacteria and *Bacteroides*, and were more often colonized with *C difficile*, when compared with vaginally born infants. More recent studies have confirmed that vaginally delivered infants acquired bacterial resembling their own mother’s vaginal microbiota whereas C-section infants harbored those similar to those found on the skin [65]. Other environmental factors can also impact upon the gut microbiome. For example antibiotic use by the infant is associated with decreased numbers of bifidobacteria and *Bacteroides*, and infants with older siblings had slightly higher numbers of bifidobacteria, compared with infants without siblings [64]. Finally as described earlier epidemiological studies have shown that children who grow up on traditional farms are protected from asthma, hay fever and allergic sensitization [56, 66]. Interestingly a number of host genes including MYD88 [67], NOD2 [68] and defensins [69] have been shown to effect the composition of the gut microbiota identifying a series of mechanisms whereby host genes and environment interact to shape the gut microbiome [70].

## Conclusions

**Fig. 1:**
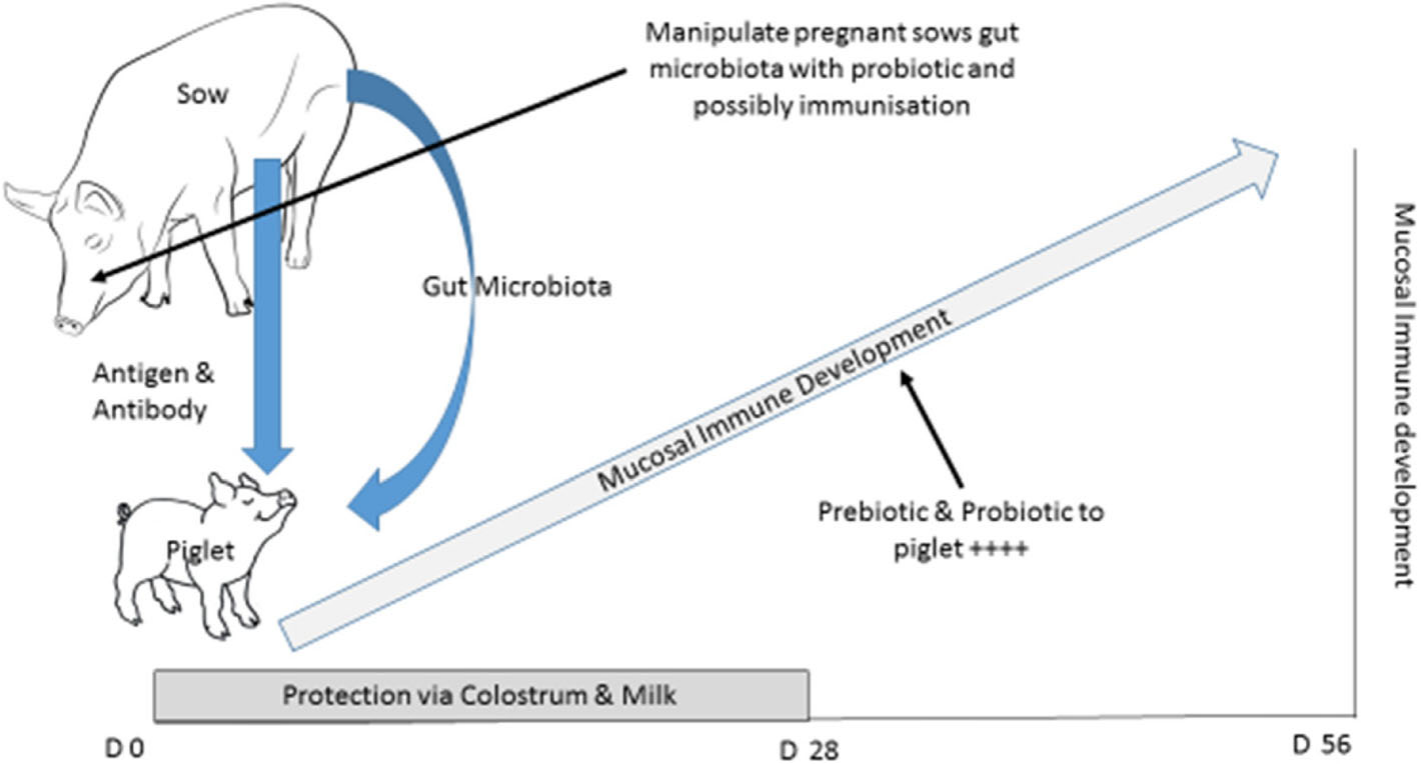
Mucosal Immune development in the young piglet. Piglet’s are born with an immature mucosal immune system which develops over the first few weeks of life following a programmed sequence. The sow to piglet interaction is pivotal to the development of the piglet’s immune system as she provides her offspring with antigen and antibody via colostrum (and milk) and gut microbiota

There is then clear evidence that at birth the mucosal immune system of piglets is immature, with maturation occurring over the first few weeks of life following a programmed sequence. At this time the piglet is highly dependent for protection from infection upon maternal colostrum and milk. Following weaning the piglet’s mucosal immune system is required to recognise and respond appropriately to both potential pathogens (to prevent fatal infectious diseases) and “harmless dietary and environmental antigens” (to avoid damaging allergic reactions). Commercially most piglets are weaned at an age when their mucosal immune system is not fully mature, underling the high morbidity and mortality resulting from post-weaning diarrhoea. Several years ago we hypothesised that an aberrant immune responses to antigens in the post-weaning diet might predispose to enteric bacterial infection and diarrhoea in the post-weaning period [41]. The sow to piglet interaction is pivotal to the development of the piglet’s immune system and occurs at multiple levels [71]. As discussed earlier host genetic factors are important in mucosal immune development but the sow also provides her offspring with antigen and antibody via colostrum (and milk) [34] and gut microbiota [58, 59] (Fig. 1). The importance of mode of maternal delivery and rearing environment have been similarly shown in studies of human infants [56, 65]. During the first few days of life the piglets’ ability to absorb dietary antigens and mount an immune response changes [72], and this response can be modulated the co-administration of specific antibody [73]. Further studies to refine this approach may provide a way to stimulate beneficial responses to dietary/environmental challenges in the post weaning period. Similarly given the pivotal role that the gut microbiota play in driving mucosal immune development and the importance of maternal microbiota, refined by environmental factors, in colonising the suckling infants gut, it might be possible to optimise the transfer of selected bacterial populations through microbial colonisation and immunisation of pregnant sows during the later stages gestation and early lactation [74].

The reduced requirement for anti-microbials in the post-weaning period would have a major public health benefit.

## Abbreviations

APC: Antigen presenting cell
CARDS: Caspase recruitment domains
CD: Cluster of differentiation
C-section: Cesarean section
DGGE: Denaturing gradient gel electrophoresis
HEPA: High-efficiency particulate arrestance
IEL: Intraepithelial lymphocyte
Ig: Immunoglobulin
IL-: Interleukin
LPS: Lipopolysaccharide
LRRs: Leucine-rich repeats
MAdCAM-1: Mucosal cell addressin molecule-1
MAPK: Mitogen-activated protein kinase
M-cell: Microfold cell
MDP: Muramyl dipeptide
MHC: Major histocompatibility complex
MIL: Mucosal immunology Langford
NBD: Nucleotide binding domain
NLRs: Nucleotide-binding oligomerisation domain (NOD)-like receptors
NOD: Nucleotide-binding oligomerisation domain
PAMPs: Pathogen-associated molecular patterns
PCA: Principal component analysis
pIgR: Polymeric immunogloblin receptor
PRRs: Pattern recognition receptors
SPF: Specific pathogen free
T-cell: Thymus derived cell
TIR: Toll/interleukin (IL)-1 receptor
TLRs: Toll-like receptors
T-regs: Foxp3^+^ regulatory T cells.
